# Stable isotope signatures reflect dietary diversity in European forest moths

**DOI:** 10.1186/s12983-016-0170-0

**Published:** 2016-08-22

**Authors:** Marc-Oliver Adams, Carlo Lutz Seifert, Lisamarie Lehner, Christine Truxa, Wolfgang Wanek, Konrad Fiedler

**Affiliations:** 1 Department of Botany and Biodiversity Research, University of Vienna, Rennweg 14, 1030 Vienna, Austria; 2 Department of Microbiology and Ecosystem Science, University of Vienna, Althanstrasse 14, 1090 Vienna, Austria; 3 Biology Center, Institute of Entomology, University of South Bohemia and Czech Academy of Sciences, Branišovska 31, 37005 Česke Budějovice, Czech Republic

**Keywords:** δ^13^C, δ^15^N, Larval diet, Trophic position

## Abstract

**Background:**

Information on larval diet of many holometabolous insects remains incomplete. Carbon (C) and nitrogen (N) stable isotope analysis in adult wing tissue can provide an efficient tool to infer such trophic relationships. The present study examines whether moth feeding guild affiliations taken from literature are reflected in isotopic signatures.

**Results:**

Non-metric multidimensional scaling and permutational analysis of variance indicate that centroids of dietary groups differ significantly. In particular, species whose larvae feed on mosses or aquatic plants deviated from those that consumed vascular land plants. Moth δ^15^N signatures spanned a broader range, and were less dependent on species identity than δ^13^C values. Comparison between moth samples and ostensible food sources revealed heterogeneity in the lichenivorous guild, indicating only *Lithosia quadra* as an obligate lichen feeder. Among root-feeding *Agrotis segetum*, some specimens appear to have developed on crop plants in forest-adjacent farm land. Reed-feeding stem-borers may partially rely on intermediary trophic levels such as fungal or bacterial growth.

**Conclusion:**

Diagnostic partitioning of moth dietary guilds based on isotopic signatures alone could not be achieved, but hypotheses on trophic relationships based on often vague literature records could be assessed with high resolution. Hence, the approach is well suited for basic categorization of moths where diet is unknown or notoriously difficult to observe (i.e. Microlepidoptera, lichen-feeders).

**Electronic supplementary material:**

The online version of this article (doi:10.1186/s12983-016-0170-0) contains supplementary material, which is available to authorized users.

## Background

Understanding the trophic structure within a given community is a vital step in understanding the underlying system as a whole. Nonetheless, dietary information at species level, especially in invertebrates, remains patchy and anecdotal in many cases. With approximately 160,000 validly described species worldwide, Lepidoptera are the second most diverse insect order after Coleoptera [1] with pronounced environmental impact, especially during their larval stages. Commonly, caterpillars are collectively perceived as herbivorous [2, 3], but in fact they span a wide range of dietary guilds from ‘classic’ herbivores, to detritivores, lichen and fungal feeders [4], and even predatory species [5, 6]. Recent case studies on caterpillar assemblages [7, 8] suggest that such ‘unusual’ feeding habits may be far more prevalent than commonly thought.

The lack of detailed dietary information is partly related to sampling difficulties: Caterpillars are typically cryptic in coloration and behaviour ([9] for comprehensive review), and often occur at low densities. Furthermore, their mere presence on a plant does not necessarily imply a trophic relationship since many species are known to move to neighbouring non-host plants for reasons of predator avoidance [10] or thermoregulation [11]. In vivo feeding trials [12] and gut content analysis e.g. [13] offer more precise insight, but these approaches are time consuming and only reveal dietary habits during a short time span prior to sampling. This may result in misleading or incomplete dietary classification as some species switch diet during their larval development, actively mix diets for nutritional balance [14], or feed opportunistically, exploiting different food sources if and when they are available [15].

Stable isotope analysis has the potential to circumvent many of these caveats. In holometabolous insects, certain organs (e.g. wings) are metabolically largely inactive following metamorphosis and thus broadly conserve the isotopic signature acquired during the larval phase ([16], but see [17]). Information on caterpillar diet can therefore be gleaned from adult specimens, which in nocturnal moths can be easily and efficiently sampled using light traps. Furthermore, carbon (C) and nitrogen (N) isotopic ratios reflect an organism’s dietary history integrated over its lifetime rather than only its most recent food source. Over recent decades, methodological advances and cost reduction have made isotope analysis a powerful and efficient tool in ecological research and trophic network analysis ([18] and citations therein), particularly with regard to arthropods where feeding habits of individual species are difficult to observe and quantify ([19–22] for comprehensive review). Research on oribatid mites [23] and springtails [24] has demonstrated the feasibility of even fine-scaled distinction between different dietary guilds based on C and N isotopic ratios. Despite such promising prospects, the use of stable isotopes in Lepidopteran nutritional ecology has so far been largely limited to a few agriculturally relevant species [16, 25–28].

In an effort to fathom the potential of stable isotope analysis in assessing caterpillar dietary guild affiliations, we here examine whether isotopic signatures of adult moths can be matched to the corresponding larval feeding guilds derived from trait information in literature.

## Methods

Moths were collected using automated light traps as part of a prior study on moth communities in the Danube floodplain forests near Vienna, Austria, between 2006 and 2008 [29, 30]. Immediately after retrieval, trap catches were stored in a freezer (-20°C) and subsequently identified to species level using standard faunal monographs. The nomenclature of moth species follows the Fauna Europaea project (http://fauna.naturkundemuseum-berlin.de). For the present study, we focused on taxa with well-documented feeding habits and sufficient abundance to allow for adequate sample size. From the available species pool, we selected 47 species from eight dietary guilds according to affiliations with larval resources, namely aquatic plants (subsequently referred to as ‘aquatic’; 2 spp.), grasses (4 spp.), herbaceous plants (‘herb’; 9 spp.), lichens (5 spp.), decaying foliage of (mostly) woody plants (‘litter’; 8 spp.), mosses (2 spp.), reeds (2 spp.), roots (1 sp.), and living foliage of woody plants (‘tree’; 14 spp.). Guild affiliation was determined based on recent comprehensive literature [31–41]. The asymmetry in the number of sample species per category is due to the uneven representation of dietary niches among temperate-zone moth assemblages, e.g. [42].

Only wing tissue was used for stable isotope analysis. Analysis was based on samples consisting of the pooled wings of three to five (depending on body size) moth specimens of one species to help level out possible variation between individuals. Each species was represented by between one and nine such samples, depending on availability of specimens, resulting in a total of 231 samples. Furthermore, we analyzed 33 potential food sources corresponding to the dietary guilds outlined above, resulting in a further 76 samples; for details see supplementary material (Additional file 1: Table S1A). Substrate samples were collected in the vicinity of the original light-trap locations in the Danube floodplain forest during the summer months of 2014. The selected plant species are ubiquitous in the study area, have been reported as part of the dietary niche of the selected moth species and can therefore be assumed to having served as potential host for these moths, even though the feeding history of the analyzed specimens was of course not known.

All insect and substrate samples were dried and loaded (*c*.1.0 mg) into pre-cleaned tin capsules for isotopic analysis; substrate samples were ground to a fine powder in a ball mill (Retsch MM2, Vienna, Austria) prior to loading. δ^13^C and δ^15^N values were quantified by continuous-flow gas isotope ratio mass spectrometry. The elemental analyzer (EA 1110, CE Instruments, Milan, Italy) was interfaced via a ConFlo II device (Finnigan MAT, Bremen, Germany) to the gas isotope ratio mass spectrometer (Delta^PLUS^, Finnigan MAT). Analyses were carried out at the Department of Microbiology and Ecosystem Science, University of Vienna. High purity CO_2_ and N_2_ reference gases were run with each analysis. Reference gases were calibrated to V-PDB (Pee Dee Belemnite) and atmospheric nitrogen (at-air) international standards using IAEA-CH-6, IAEA-CH-7 for δ^13^C, and IAEA-N-1, IAEA-N-2 and IAEA-NO-3 for δ^15^N (IAEA, Vienna, Austria).

Due to conspicuous differences in δ^13^C and δ^15^N values between *Lithosia quadra* and other nominal members of the lichen-feeding guild, the decision was made to treat this species as a separate category. In order to discern whether or not moth dietary guilds can be differentiated based on their isotopic signatures, we conducted non-metric multi-dimensional scaling (NMDS) of the Euclidean distance matrix between all individual moth samples, calculated from their combined δ^13^C and δ^15^N values (scaled to a mean of zero and a variance of one prior to analysis). Differentiation of group centroids within the resulting ordigram was tested using permutational analysis of variance (PERMANOVA; 10,000 iterations) and subsequent pair-wise post hoc comparisons using a permutational *t*-test, both implemented in PERMANOVA+ [43] for PRIMER7 [44]. Group centroid differences were further visualized by means of bootstrap averaging, likewise available in PRIMER7. Bootstrap averaging is based on repeated resampling (with replacement, 50 iterations) from the original dataset; the average values are then visualized in a metric multidimensional scaling plot (MMDS), using as many dimensions as needed to closely match the original distance matrix (correlation coefficient of *rho* = 0.99; in our case *m* = 4 dimensions turned out to be sufficient).

In addition, δ^13^C and δ^15^N values of moth samples were separately analyzed using generalized linear mixed effects models (GLMMs) with dietary guild as predictor and including species identity as random factor. All models assumed a Gaussian distribution and adequate model fit was assured by assessing Q-Q-plots and histograms of the residuals. *P*-values are based on the Satterthwaite approximation of degrees of freedom. Model fit was calculated as marginal (excluding the random factor) and conditional (including the random factor) *R*^2^ following Nakagawa and Schielzeth [45]. To pinpoint differences between individual feeding guilds, we conducted pair-wise post hoc comparisons using Tukey’s HSD test. All calculations were carried out using the R framework for statistical computing [46]. δ^13^C and δ^15^N values of moth dietary guilds and the corresponding food substrate were also visualized using a bi-plot. Species-level means can be found in the supplementary material (Additional file 1: Figures S1A and S2A).

## Results

**Fig. 1:**
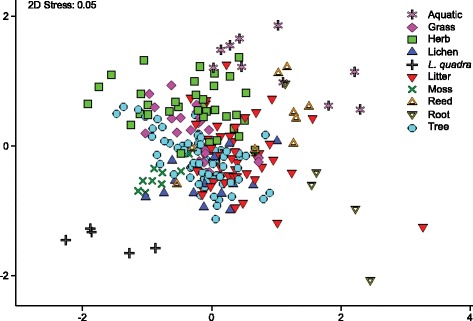
Non-metric multi-dimensional scaling plot (NMDS) of Euclidean distances between moth feeding guilds based on δ^15^N and δ^13^C values. Symbols represent individual samples of the respective moth guild

**Fig. 2:**
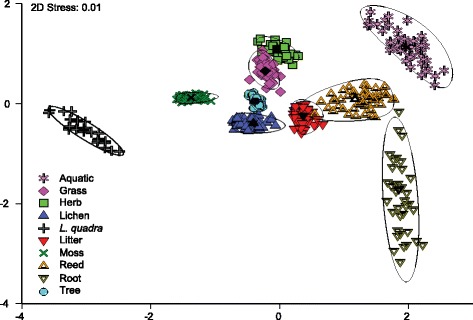
Metric multi-dimensional scaling plot (MMDS) of bootstrap averages (50 repetitions). Individual repetitions are based on random draw and replacement of samples from the original dataset. Colour symbols represent the group centroids of respective dietary guilds for each repetition; black symbols the overall centroids across all repetitions. Boundary lines approximate 95 % confidence regions

Non-metric multi-dimensional scaling (NMDS) of Euclidean distances based on combined evidence from δ^13^C and δ^15^N suggests that dietary guilds among moths can be well distinguished based on stable isotope analysis. Particularly, *L. quadra*, aquatic species, as well as root-and moss-feeders are clearly positioned away from foliage-feeders in reduced ordination space (see Fig. 1). In contrast, moths with litter-feeding larvae show substantial variation in their isotopic signatures. Grass, herb and tree feeders form data clouds that largely overlap when looking at individual samples. However, a PERMANOVA confirmed that highly significant differences exist between group centroids (*df* = 9, pseudo-*F* = 26.2, *p* < 0.001), and subsequent pair-wise post hoc comparisons showed that indeed all centroids differ significantly from one another (see Table 1). This finding is further corroborated by bootstrap averaging which shows group centroids to be reliably different between individual feeding guilds (see Fig. 2).

**Table 1:** PERMANOVA post hoc pair-wise comparisons of dietary guilds based on 10,000 permutations

	Aquatic	Grass	Herb	Lichen	*L. quadra*	Litter	Moss	Reed	Root
Grass	5.76 ***								
Herb	5.02 ***	1.80 *							
Lichen	6.94 ***	3.99 ***	5.30 ***						
*L. quadra*	7.97 ***	7.59 ***	6.90 ***	6.36 ***					
Litter	5.20 ***	3.97 ***	5.91 ***	2.77 ***	6.67 ***				
Moss	7.36 ***	4.37 ***	4.53 ***	3.64 ***	5.45 ***	4.71 ***			
Reed	2.23 **	4.02 ***	4.02 ***	4.29 ***	6.78 ***	2.16 *	5.68 ***		
Root	3.51 **	6.25 ***	6.25 ***	4.79 ***	5.97 **	3.83 ***	6.35 ***	2.48 **	
Tree	8.14 ***	2.91 **	5.79 ***	1.89 *	6.82 ***	4.40 ***	3.37 ***	4.98 ***	6.64 ***

Values represent *t*-scores,* symbols the respective significance level of the comparison

Significance codes: *p* ≤ 0.001 ‘***’; *p* ≤ 0.01 ‘**’; *p* ≤ 0.05 ‘*’

**Fig. 3:**
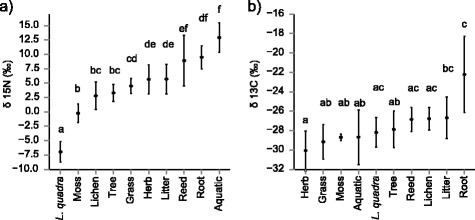
The plots depict mean **a** δ^15^N and **b** δ^13^C values across moth feeding guilds. Whiskers represent the respective standard deviation. For illustrative purposes, guilds are arranged from lowest to highest mean and the order therefore differs between graphs. The compact letter display is based on pair-wise post hoc comparison with Tukey’s HSD correction. Different letters indicate a significant difference between the respective feeding guilds

GLMM analysis of moth samples also revealed significant differences between dietary guilds on the level of individual isotopic measures. Differentiation was most pronounced for the δ^15^N values (*df* = 9; *F* = 22.861, *p* < 0.001; *R*^2^_marginal_: 0.642; *R*^2^_conditional_: 0.731) with individual sample values spanning a range from -14.6 ‰ to 17.2 ‰. Nitrogen isotopic signatures were highest among reed-and root-feeding taxa, and particularly so among aquatic species. δ^15^N values were lowest for *L. quadra* and notably different from other members of the lichen-feeding guild (see Fig. 3a).

δ^13^C values of individual samples ranged between-36.5‰ and-15.9‰. While statistical analysis showed overall significant differences between feeding guilds (*df* = 9; *F* = 3.859, *p* < 0.01; *R*^2^_marginal_: 0.318; *R*^2^_conditional_: 0.668), most species fell within a fairly narrow band with mean values between -30‰ and -27‰. The root-feeding guild represented by one single species (*Agrotis segetum*) revealed the only divergence from this pattern with an average value of-22‰ and considerable variance between individual samples (see Fig. 3b). In contrast to nitrogen isotopic signatures, the random factor (i.e. moth species) accounted for more than half of the model’s explanatory power in the analysis of δ^13^C values as indicated by the large difference between marginal and conditional *R*^2^.

**Fig. 4:**
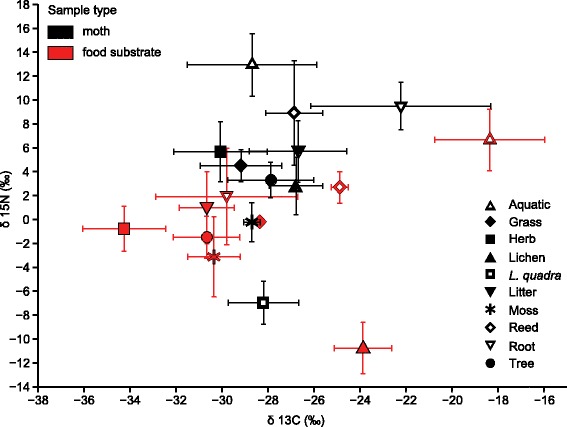
Biplot of δ^13^C and δ^15^N values for moth dietary guilds (black symbols) and the corresponding food substrates (red symbols). Symbols depict the mean of across all members of the respective guild, whiskers represent the standard deviation

The δ^13^C × δ^15^N biplot illustrates the position of moth dietary guilds relative to their nominal food substrates (see Fig. 4). All moths showed clear ^15^N enrichment (higher δ^15^N values) relative to their nominal food sources, but the magnitude of this varied between guilds. Moss-feeding Lepidoptera and *L. quadra* displayed enrichments of roughly 3–4‰ relative to their corresponding substrate samples, while all other groups deviated to a greater extent with the highest discrepancy observed between nominal lichen-feeders and their prospective food source (13.6‰ ^15^N enrichment; see Table 2). With regard to carbon isotopic signatures, dietary guilds are divided between groups that displayed lower δ^13^C values compared to their presumed substrate, and those that are characterized by relatively higher values. The former category is comprised of aquatic species, and those feeding on herbs, lichens, and reeds, respectively; the latter includes grass-, litter-, moss-, root- and tree-feeding taxa. Notably, the aquatic and root-feeding guilds showed considerable deviation in carbon isotopic fractionation relative to their prospective food source (see Table 2)

**Table 2:** Mean δ^13^C and δ^15^N values for each moth dietary guild and the corresponding substrate group

	δ ^15^N			δ ^13^C		
	Moth	Substrate	Δ	Moth	Substrate	Δ
Aquatic	12.9 (±2.6)	6.7 (± 2.6)	6.3	−28.7 (± 2.8)	−18.4 (± 2.4)	−10.3
Herb	4.5 (± 1.3)	−0.2 (-n.a -)	4.7	−29.2 (± 1.8)	−28.4 (-n.a.-)	−0.8
Grass	5.7 (± 2.5)	−0.8 (± 1.9)	6.4	−30.1 (± 2.0)	−34.3 (± 1.8)	4.2
Lichen	2.8 (± 2.4)	−10.8 (± 2.2)	13.6	−26.8 (± 1.2)	−23.9 (± 1.3)	−2.9
*L. quadra*	−7.0 (± 1.8)	−10.8 (± 2.2)	3.8	−28.2 (± 1.5)	−23.8 (± 1.3)	−4.3
Litter	5.7 (± 2.6)	1.0 (± 3.0)	4.7	−26.7 (± 2.1)	−30.7 (± 1.2)	4.0
Moss	−0.2 (± 1.6)	−3.1 (± 3.4)	2.9	−28.7 (± 0.4)	−30.3 (± 1.1)	1.7
Reed	8.9 (± 4.4)	2.7 (± 1.3)	6.2	−26.9 (± 1.2)	−24.9 (± 0.4)	−2.0
Root	9.5 (± 2.0)	1.9 (± 4.1)	7.6	−22.2 (± 3.9)	−29.8 (± 3.1)	7.6
Tree	3.3 (± 1.5)	−1.5 (± 1.8)	4.8	−27.9 (± 1.9)	−30.7 (± 1.4)	2.8

Values correspond to the biplot in Fig. 2. Values in parentheses provide the standard deviations (SD). In the case of grasses, only one substrate sample was analyzed and SD could therefore not be calculated. For both isotopes, the discrepancy between moth means and substrate means across samples is included (Δ values, equivalent to the trophic level enrichment, TLE)

.

## Discussion

Species with aquatic caterpillars, *L. quadra*, moss-and root-feeders, respectively, form clearly delineated groups that stand apart from the other samples in an NMDS ordination. The remaining dietary guilds show a greater degree of overlap, and also more substantial variation between samples and species, which would render a diagnostic guild assignment based solely on isotopic ratios difficult for the present dataset. At the same time, however, concomitant PERMANOVA indicates that respective guild centroids are all significantly different from one another, suggesting that the groups could potentially be resolved. Previous studies showing good resolution between closely related feeding guilds (e.g. primary and secondary decomposers; [23]) are characterized by larger sample size and a greater homogeneity of samples. For example, small body size of the focal organisms (i.e. oribatid mites) combined with minimum mass required for isotope analysis meant that each sample typically comprised 3–255 individuals [47] while our samples contained tissue from at most five specimens. Given the exploratory nature of the present study and the inherent complexity underlying the isotopic composition of an organism, it is perhaps unsurprising that not all dietary guilds could be unambiguously resolved by stable isotope analysis alone. Like any tool, it needs to be honed for its task. Homogenization of samples and a greater overall sample size in analogy to [47], as well as a closer understanding of the physiological determinants of moth isotopic composition would likely allow for an even higher discriminative power between dietary guilds. Along this vein, however, the present data indicated a number of interesting patterns which may serve to guide further scrutiny of moth isotopic composition.

### Nitrogen

δ^15^N signatures spanned a broader range of values and showed a closer link to feeding guilds than δ^13^C patterns. Primary consumers are expected to show δ^15^N values that are approximately 3–4‰ higher than those of their respective host plants [21, 48], although lower values of enrichment have also been reported [49]. This is due to preferential excretion of isotopically lighter N compounds, causing ^15^N enrichment of the consumer relative to its diet, i.e. trophic level enrichment of N isotopes. Substantial deviations from expected enrichment increments may suggest a nitrogen source other than the presumed host substrate or a higher-level trophic link. Reliance on unexpected hosts is exemplified by the lichenivorous guild in the present dataset: At an average of +3.8‰, Δ^15^N values for *L. quadra* were within the expected range for a primary consumer of lichens (−10.8‰), while other representatives of the guild (i.e. *Eilema griseola*, *E. lurideola*, *Miltochrista miniata*, and *Pelosia muscerda*) deviated by more than 13‰ from their nominal substrate. Although nitrogen isotope signatures of lichens have been shown to vary with soil condition [50] and atmospheric N concentrations [51], the magnitude of the observed discrepancy suggests that among the examined species, only *L. quadra* is obligately lichenivorous in the study region. The other taxa appear to rely to a large degree on other sources (e.g. free-living algae, mosses), as has been occasionally reported for members of the Lithosiini tribe [33, 52]. Given the large discrepancy between obligate and facultative lichen feeders, stable isotope analysis thus represents a convenient way to discern and revise larval diet for a moth guild that is notoriously difficult to raise in captivity. The litter-feeding guild, on the other hand, is an example where humification processes and microbial activity in decaying foliage introduce additional trophic levels between the host-plant and the consumer, thus explaining the elevated ^15^N enrichments as well as the large variance observed in detritivorous species [22].

Surprisingly, the moderate trophic level enrichment in ^15^N observed for litter-feeders compared to their ostensible substrate was small compared to the values recorded for taxa with aquatic caterpillars, as well as reed-and root-feeding species. Previous studies have shown elevated δ^15^N values in root tissue e.g. [53], and in aquatic versus terrestrial plants [54, 55], but our moth samples exceeded that baseline by approximately +6–8‰, thus suggesting something other than a simple trophic link. Larvae of the two examined reed-feeding species (*Phragmataecia castaneae* and *Mythimna obsoleta*) bore into the stem of their host and feed internally. The activity of plant-boring insects is sometimes associated with the development of secondary fungal or bacterial infections in the affected host [56, 57]. Increased ^15^N enrichment of reed-boring taxa may thus be related to the intentional or inadvertent consumption of intermediary trophic levels. In the present context, this might be favoured by protracted larval development of the sample species (e.g. 2 years in *P. castaneae*) which would allow such secondary microbial colonists ample time to develop, as well as by the damp, eutrophic nature of floodplain marshland which is also likely to favour fungal and bacterial growth.

In our sample, the root-feeding moth guild was represented by only one single species that was sufficiently abundant for analysis. The highly polyphagous *Agrotis segetum* is also known as a serious pest in commercial corn (*Zea mays*) and vegetable fields [35, 58]. The use of manure rather than synthetic fertilizer has been shown to shift the nitrogen isotopic ratio of crops towards the heavier isotope [59]. Consequently, elevated δ^15^N levels in *A. segetum* likely reflect agricultural management practices in the larval habitat, rather than being representative of the guild as a whole. Truxa and Fiedler [30] regarded *A. segetum* as the most abundant stray species in the moth communities of the floodplain forests in eastern Austria. The aberrant isotopic signature reported here indeed supports the notion that most of these moths did not develop in the forest habitat (a nature reserve) where they had been sampled, but rather originated from the surrounding agricultural landscape.

Moth species feeding on grasses, herbs and foliage of woody vegetation likewise displayed fairly high δ^15^N enrichments relative to their nominal hosts, which is both unexpected and difficult to explain. Although unavoidable under the circumstances, the delay between moth collection and substrate sampling could have played a role in the present study. Isotopic composition has been shown to vary between different types of tissue in a given plant [60, 61], as a result of stress [62], or due to seasonal [63] and even diurnal rhythms [64]. Moreover, mobility of adult animals, the polyphagy of most of our folivorous study species and the fairly high heterogeneity of habitat increase the likelihood that sampled food substrate and factual food sources differ with regard to key environmental parameters and isotope composition. The moisture content of soils has an impact on nitrification and denitrification processes [65] and consequently on the ^15^N enrichment of available soil N and plants – an effect that is undoubtedly relevant in periodically inundated floodplain forest. Similarly, other environmental parameters [66, 67] and canopy openness [68] can affect isotopic fractionation in soil-plant systems and, by extension, the isotopic composition of associated consumers.

Lastly, there have been observations of a systematic shift in δ^15^N signatures during the process of metamorphosis [17]. In contrast to our study, Tibbets et al. sampled adult moths as a whole immediately upon emergence. Given that isotopic fractionation has been shown to differ between tissue types [69], and that wing primordia are already present in caterpillars prior to pupation [70] it is not unreasonable to assume that larval signature is more faithfully preserved in some body parts than in others (but see [27]). All moth species selected for analyses are characterized by short adult life-spans (usually less than 1 month) and sample selection focused on undamaged (and hence younger) specimens. This was aimed to further limit the extent of post-metamorphosis changes in isotopic fractions. On the whole, there has been little research on this issue, but the results presented here and in other studies [16, 25] suggest that isotopic signatures in adult wing tissue are sufficiently stable to allow inference of larval diet. Addressing such factors in detail was beyond the scope of the present study, but would allow valuable insights for future research.

### Carbon

δ^13^C signatures were for the most part fairly similar between moth samples, and variation was as much a function of dietary guild affiliation as of species identity. ^13^C fractionation varies between photosynthetic pathways and carbon isotopic ratios therefore predominantly indicate the primary producer within a given food web. Since temperate ecosystems are dominated by C_3_ plants, respective isotope ratios typically fall within a narrow range and show only minor changes across trophic levels [71]. Accordingly, our data showed similar δ^13^C values among dietary guilds, with the only notable exception of the root-feeding *A. segetum* which displayed significantly higher δ^13^C signatures. As pointed out before, *A. segetum* feeds not only on native European plants but also on maize. Like all C_4_ plants, *Z. mays* is characterized by lower carbon isotopic fractionation and more positive δ^13^C values (−12 to−20‰) relative to C_3_ plants (−25 to−32‰) [72]. Elevated δ^13^C values thus lend further support for maize crops as a host for representatives of this moth species in the present setting. At the same time, considerable variation among moth samples suggests that not all collected specimens of this highly polyphagous species made use of this food source.

All other dietary guilds were fairly similar to each other with regard to their δ^13^C values, but showed in part considerable deviation from the isotopic composition of their ostensible host substrate. ^13^C enrichment of litter-feeding taxa is in line with previous studies that have linked enrichment of up to 3‰ to the activity of saprotrophic fungi in decaying plant matter (for comprehensive review [22, 73]).

## Conclusions

The present study was exploratory in nature, since (in contrast to other litter-dwelling insect groups: e.g. springtails and oribatid mites) no multi-species analyses of stable isotope signatures have thus far been published for Lepidopterans. Even the present dataset did not allow unambiguous separation of dietary guilds based on carbon and nitrogen isotopic fractions alone. Our results strongly suggest that better guild resolution may potentially be achieved with larger sample sizes and more detailed knowledge of underlying determinants of isotopic fractionation in plants and animals. Even at this relatively low resolution, however, the approach emerged as well suited to broadly confirm hypotheses on larval trophic relationships based on often vague anecdotal records in literature. We therefore conclude that this approach would also be highly useful to expand insight into nutritional ecology for groups where little is known about feeding habits (i.e. Microlepidoptera, tropical taxa) or where the exact source of nutrition is difficult to discern through rearing experiments (i.e. lichen-or detritus-feeders).

## Additional file

Additional file 1: Supplementary material. **Table S1A.** Number of substrate and moth samples taken per species and feeding guild. To level out variability between moth individuals, each sample consisted of the wings of 3–5 moth specimens (depending on body size of the species), so that each sample had a mass of 1–2 mg. The nomenclature of moths follows Fauna Europaea (http://fauna.naturkundemuseum-berlin.de). **Figure S1A.** Fraction of 15N relative to 14N for each moth species. Taxa are ordered by feeding guild and red coloration represents the overall mean for the respective guild aggregated across constituent species. Symbols represent the mean and whiskers the standard deviation for each group. Among the lichen-feeding species, *L. quadra* was evaluated separately from the remaining taxa due to its deviant nitrogen signature. **Figure S2A.** Fraction of 13C relative to 12C for each moth species. Taxa are ordered by feeding guild and red coloration represents the overall mean for the respective guild aggregated across constituent species. Symbols represent the mean and whiskers the standard deviation for each group. Among the lichen-feeding species, *L. quadra* was evaluated separately from the remaining taxa due to its deviant nitrogen signature. (PDF 860 kb)

## Abbreviations

C: Chemical symbol for carbon
IAEA-CH-6: Reference standard for ^13^C/^12^C ratios derived from sucrose and provided by the international atomic energy agency (IAEA)
IAEA-CH-7: Reference standard for ^13^C/^12^C ratios derived from polyethylene and provided by the international atomic energy agency (IAEA)
IAEA-N-1: Reference standard for ^15^N/^14^N ratios derived from ammonium sulfate and provided by the international atomic energy agency (IAEA)
IAEA-N-2: Reference standard for ^15^N/^14^N ratios derived from ammonium sulfate and provided by the international atomic energy agency (IAEA)
IAEA-NO-3: Reference standard for ^15^N/^14^N ratios derived from potassium nitrate and provided by the international atomic energy agency (IAEA)
MMDS: Metric multi-dimensional scaling
N: Chemical symbol for nitrogen
NMDS: Non-metric multi-dimensional scaling
SD: Standard deviation
TLE: Trophic level enrichment
δ ^13^C: Shift in the ^13^C/^12^C ratio of the sample relative to the reference standard (i.e. Pee Dee Belemnite)
δ ^15^N: Shift in the ^15^N/^14^N ratio of the sample relative to the reference standard (i.e. atmospheric nitrogen)
