# Supplementary material for: Stable isotope signatures reflect dietary diversity in European forest moths
Source: Front Zool. 2016 Aug 22;13(1):37. doi: 10.1186/s12983-016-0170-0 (PMC4994389; doi:10.1186/s12983-016-0170-0)
Supplement: Supplementary file 1 — Supplementary material. Table S1A. Number of substrate and moth samples taken per species and feeding guild. To level out variability between moth individuals, each sample consisted of the wings of 3–5 moth specimens (depending on body size of the species), so that each sample had a mass of 1–2 mg. The nomenclature of moths follows Fauna Europaea (http://fauna.naturkundemuseum-berlin.de). Figure S1A. Fraction of 15N relative to 14N for each moth species. Taxa are ordered by feeding guild and red coloration represents the overall mean for the respective guild aggregated across constituent species. Symbols represent the mean and whiskers the standard deviation for each group. Among the lichen-feeding species, L. quadra was evaluated separately from the remaining taxa due to its deviant nitrogen signature. Figure S2A. Fraction of 13C relative to 12C for each moth species. Taxa are ordered by feeding guild and red coloration represents the overall mean for the respective guild aggregated across constituent species. Symbols represent the mean and whiskers the standard deviation for each group. Among the lichen-feeding species, L. quadra was evaluated separately from the remaining taxa due to its deviant nitrogen signature. (PDF 860 kb) [file 12983_2016_170_MOESM1_ESM.pdf]

Table A1 Number of substrate and moth samples taken per species and feeding guild. To level out variability between moth individuals, each sample consisted of the wings of 3-5 moth specimens (depending on body size of the species), so that each sample had a mass of 1-2 mg. The nomenclature of moths follows Fauna Europaea (http://fauna.naturkundemuseum-berlin.de).

Substrate samples

**Aquatic (living foliage)**		
Haloragaceae	*Myriophyllum spicatum*	3
Potamogetonaceae	*Potamogeton pectinatus*	3
**Grass (living foliage)**		
Poaceae	*Phalaris arundinacea*	1
**Herb (living foliage)**		
Balsaminaceae	*Impatiens parviflora*	3
Lamiaceae	*Stachys sylvatica*	3
Polygonaceae	*Rumex sanguineus*	3
Urticaceae	*Urtica dioica*	1
**Lichen**		
Physciaceae	*Physcia adscendens*	4
Physciaceae	*Physcia* sp.2	2
Teloschistaceae	*Xanthoria parietina*	6
**Moss**		
Lembophyllaceae	*Isothecium alopecuroides*	3
n.a.	Bryophyta sp.2	1
**Reed (living foliage)**		
Poaceae	*Phragmites australis*	3
**Root**		
Poaceae	*Phragmites australis*	3
Polygonaceae	*Rumex sanguineus*	3
Urticaceae	*Urtica dioica*	3
**Litter**		
Fagaceae	*Quercus robur*	1
Rosaceae	*Prunus padus*	1
Salicaceae	*Populus alba*	3
Salicaceae	*Populus nigra*	1
Salicaceae	*Salix alba*	1
Sapindaceae	*Acer campestre*	3
Ulmaceae	*Ulmus laevis*	3
**Tree (Woody plants; living foliage)**		
Betulaceae	*Corylus avellana*	3
Celastraceae	*Euonymus europaeus*	1
Cornaceae	*Cornus sanguinea*	1
Fagaceae	*Quercus robur*	1
Ranunculaceae	*Clematis vitalba*	1
Rosaceae	*Prunus spinosa*	1
Rosaceae	*Rubus caesius*	3
Salicaceae	*Populus alba*	3
Sapindaceae	*Acer campestre*	1
Sapindaceae	*Acer pseudoplatanus*	3

Moth samples

**Aquatic (living foliage)**		
**Crambidae**	*Cataclysta lemnata*	5
**Crambidae**	*Parapoynx stratiotata*	5
**Grass (living foliage)**		
**Crambidae**	*Agriphila inquinatella*	4
**Erebidae**	*Rivula sericealis*	7
**Noctuidae**	*Mythimna albipuncta*	5
**Noctuidae**	*Protodeltote pygarga*	5
**Herb (living foliage)**		
**Noctuidae**	*Atypha pulmonaris*	5
**Geometridae**	*Ecliptopera silaceata*	5
**Erebidae**	*Hypena proboscidalis*	7
**Erebidae**	*Phragmatobia fuliginosa*	1
**Crambidae**	*Pleuroptya ruralis*	5
**Erebidae**	*Spilosoma lubricipeda*	2
**Erebidae**	*Spilosoma lutea*	7
**Geometridae**	*Timandra comae*	2
**Noctuidae**	*Xestia c-nigrum*	5
**Lichen**		
**Erebidae**	*Eilema griseola*	9
**Erebidae**	*Eilema lurideola*	1
**Erebidae**	*Miltochrista miniata*	1
**Erebidae**	*Pelosia muscerda*	6
**Erebidae**	*Lithosia quadra*	5
**Litter**		
**Erebidae**	*Herminia grisealis*	7
**Erebidae**	*Herminia tarsicrinalis*	5
**Erebidae**	*Trisateles emortualis*	7
**Pyralidae**	*Galleria mellonella*	1
**Geometridae**	*Idaea aversata*	8
**Geometridae**	*Idaea biselata*	7
**Geometridae**	*Idaea dimidiata*	7
**Pyralidae**	*Endotricha flammealis*	5
**Moss**		
**Crambidae**	*Catoptria falsella*	4
**Crambidae**	*Catoptria verellus*	5
**Reed (living foliage)**		
**Cossidae**	*Phragmataecia castaneae*	5
**Noctuidae**	*Mythimna obsoleta*	5
**Root**		
**Noctuidae**	*Agrotis segetum*	5
**Tree (Woody plants; living foliage)**		
**Noctuidae**	*Cosmia trapezina*	5
**Noctuidae**	*Eupsilia transversa*	1
**Geometridae**	*Euchoeca nebulata*	5
**Geometridae**	*Lomaspilis marginata*	5
**Geometridae**	*Plemyria rubiginata*	5
**Notodontidae**	*Pheosia tremula*	5
**Geometridae**	*Campaea margaritaria*	6
**Geometridae**	*Cyclophora annularia*	6
**Geometridae**	*Hemistola chrysoprasaria*	5
**Geometridae**	*Ligdia adustata*	5
**Geometridae**	*Melanthia procellata*	5
**Geometridae**	*Selenia tetralunaria*	5
**Notodontidae**	*Ptilophora plumigera*	5
**Notodontidae**	*Stauropus fagi*	5

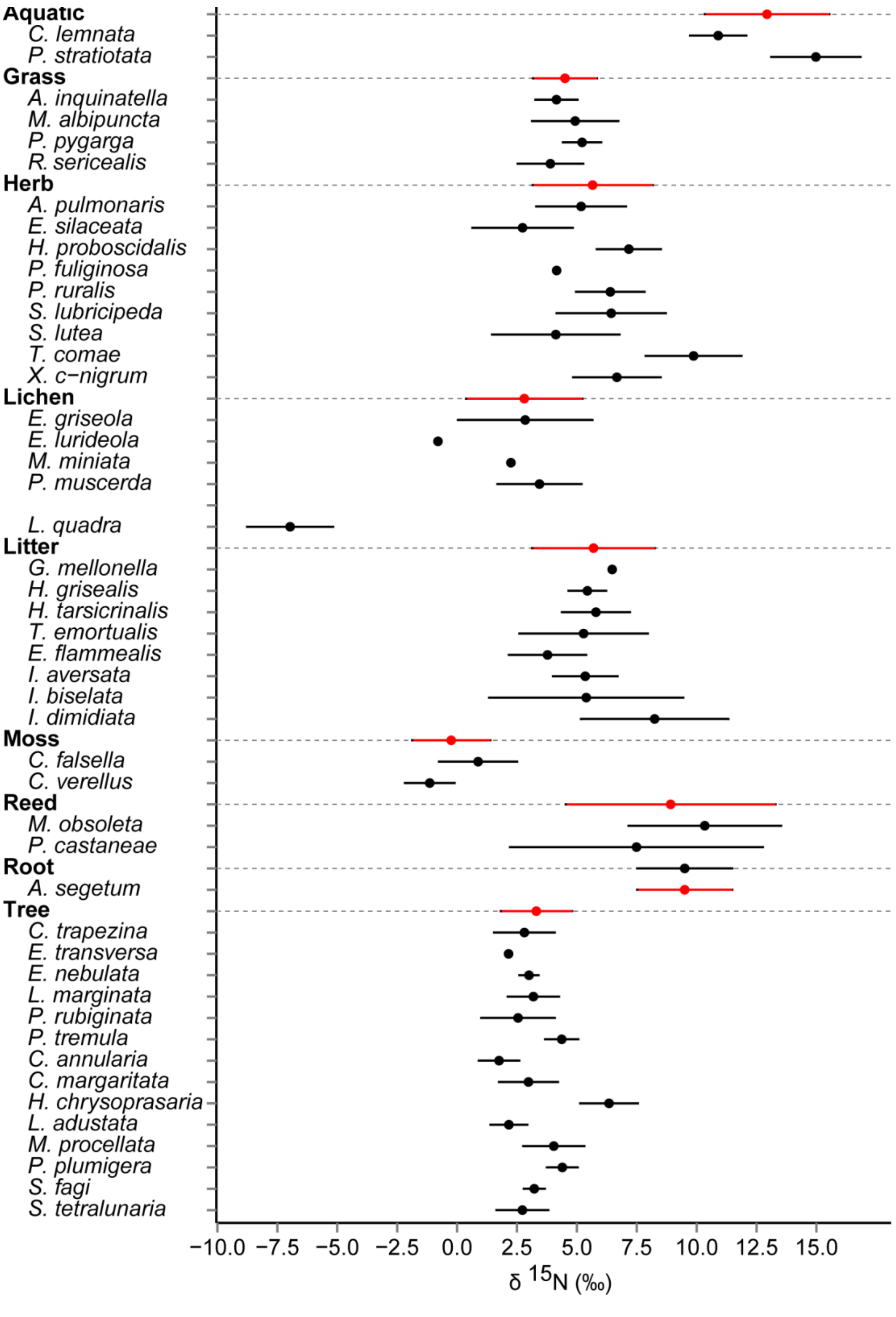

Fig. A1 Fraction of ^{15}N relative to ^{14}N for each moth species. Taxa are ordered by feeding guild and red coloration represents the overall mean for the respective guild aggregated across constituent species. Symbols represent the mean and whiskers the standard deviation for each group. Among the lichen-feeding species, *L. quadra* was evaluated separately from the remaining taxa due to its deviant nitrogen signature.

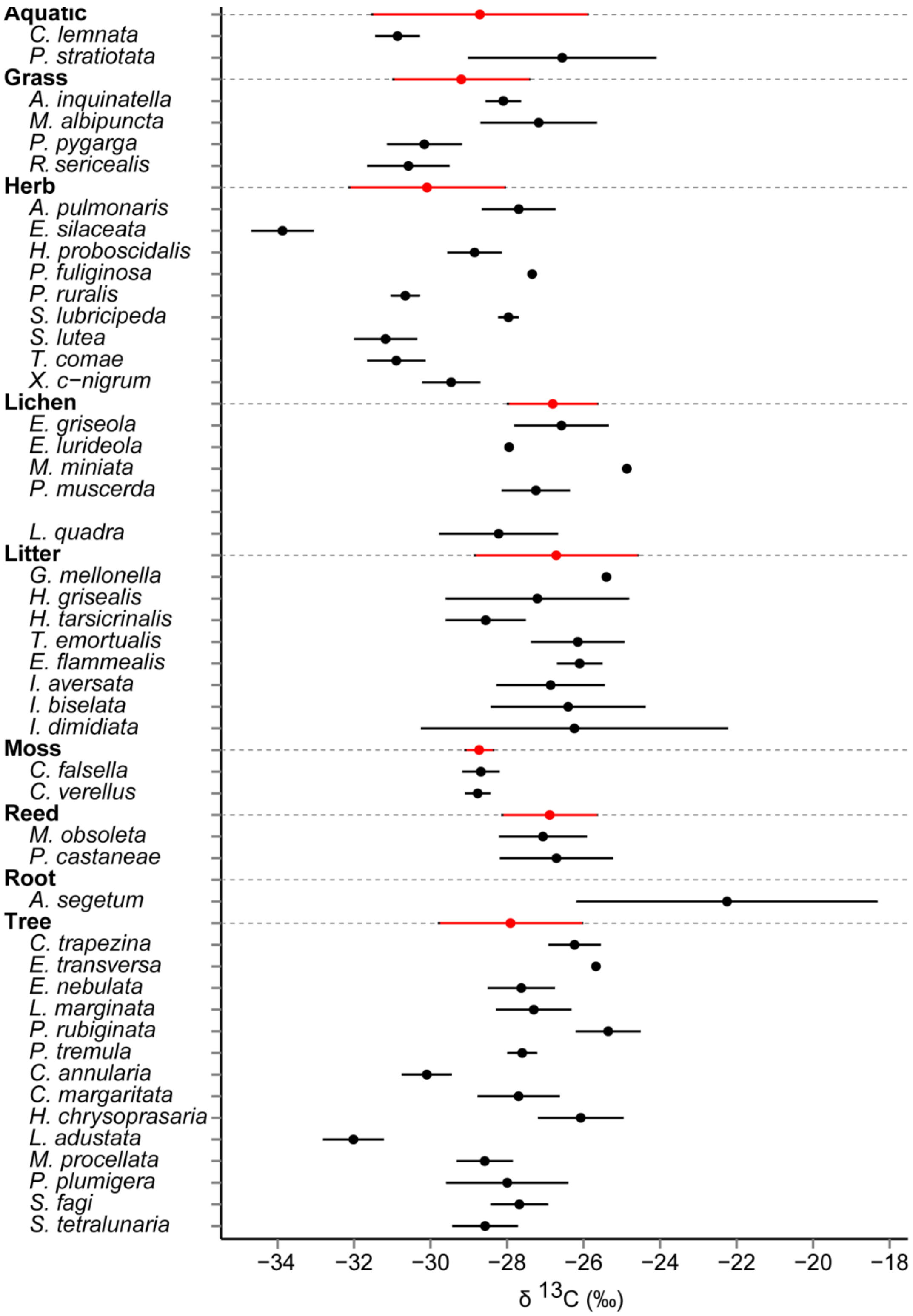

Fig. A2 Fraction of ^{13}C relative to ^{12}C for each moth species. Taxa are ordered by feeding guild and red coloration represents the overall mean for the respective guild aggregated across constituent species. Symbols represent the mean and whiskers the standard deviation for each group. Among the lichen-feeding species, *L. quadra* was evaluated separately from the remaining taxa due to its deviant nitrogen signature.
